# Functional Antimicrobial Surface Coatings Deposited onto Nanostructured 316L Food-Grade Stainless Steel

**DOI:** 10.3390/nano11041055

**Published:** 2021-04-20

**Authors:** A. Silvia González, Angela Riego, Victor Vega, Javier García, Serena Galié, Ignacio Gutiérrez del Río, Maria del Valle Martínez de Yuso, Claudio Jesús Villar, Felipe Lombó, Victor Manuel De la Prida

**Affiliations:** 1 Departmano de Física, Facultad de Ciencias, Universidad de Oviedo, C/ Federico García Lorca nº 18, 33007 Oviedo, Spain; vegavictor@uniovi.es (V.V.); garciafjavier@uniovi.es (J.G.); vmpp@uniovi.es (V.M.D.l.P.); 2 Research Unit “Biotechnology in Nutraceuticals and Bioactive Compounds—BIONUC”, Departmano de Biología Funcional, Área de Microbiología, Universidad de Oviedo, Avda. Julián Clavería 7, 33006 Oviedo, Spain; angelarg@usal.es (A.R.); serena.galie@studio.unibo.it (S.G.); nachogutiem@gmail.com (I.G.d.R.); cjvg@uniovi.es (C.J.V.); lombofelipe@uniovi.es (F.L.); 3 IUOPA (Instituto Universitario de Oncología del Principado de Asturias), 33006 Oviedo, Spain; 4 ISPA (Instituto de Investigación Sanitaria del Principado de Asturias), 33011 Oviedo, Spain; 5 Laboratorio de Membranas Nanoporosas, Edificio de Servicios Científico Técnicos “Severo Ochoa”, Universidad de Oviedo, C/ Fernando Bonguera s/n, 33006 Oviedo, Spain; 6 Servicios Centrales de Investigación, Universidad de Málaga, E-29071 Málaga, Spain; mvyuso@uma.es

**Keywords:** antimicrobial activity, stainless steel pipelines, atomic layer deposition, electroless, coatings, surface functionalization, biofilm

## Abstract

In our study, we demonstrated the performance of antimicrobial coatings on properly functionalized and nanostructured 316L food-grade stainless steel pipelines. For the fabrication of these functional coatings, we employed facile and low-cost electrochemical techniques and surface modification processes. The development of a nanoporous structure on the 316L stainless steel surface was performed by following an electropolishing process in an electrolytic bath, at a constant anodic voltage of 40 V for 10 min, while the temperature was maintained between 0 and 10 °C. Subsequently, we incorporated on this nanostructure additional coatings with antimicrobial and bactericide properties, such as Ag nanoparticles, Ag films, or TiO_2_ thin layers. These functional coatings were grown on the nanostructured substrate by following electroless process, electrochemical deposition, and atomic layer deposition (ALD) techniques. Then, we analyzed the antimicrobial efficiency of these functionalized materials against different biofilms types (*Candida parapsilosis, Escherichia coli, Pseudomonas aeruginosa, Staphylococcus aureus*, and *Staphylococcus epidermidis*). The results of the present study demonstrate that the nanostructuring and surface functionalization processes constitute a promising route to fabricate novel functional materials exhibiting highly efficient antimicrobial features. In fact, we have shown that our use of an appropriated association of TiO_2_ layer and Ag nanoparticle coatings over the nanostructured 316L stainless steel exhibited an excellent antimicrobial behavior for all biofilms examined.

## 1. Introduction

**Figure 1:**
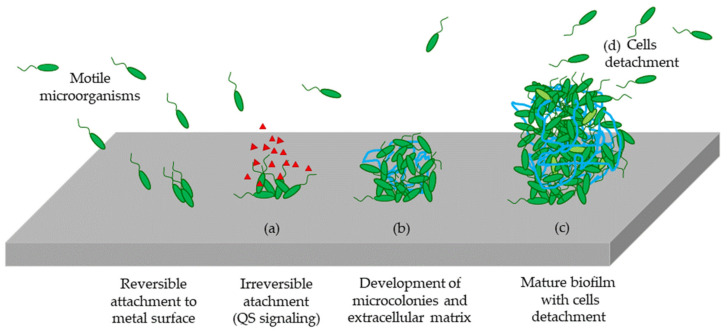
Schematic representation of a biofilm life cycle. (**a**) Bacteria attach to a surface and colonize it, (**b**) bacteria begin to produce slimy EPSs and they colonize the surface, (**c**) development of a complex three-dimensional structure, and (**d**) detachment of the biofilm and spreading. Image is self-created.

Stainless steel manufacturing can be considered as the measure of progress in modern civilization, being visibly and invisibly omnipresent in everyone’s daily life. Nowadays, stainless steel products play a key role in many industries, particularly in food and agriculture sectors, where it has become an essential material that is employed in pipeline fabrication [1]. Although stainless steel is an ideal material for manufacturing surfaces and equipment due to its physical-chemical stability and high corrosion resistance, there are many reports referring to problems in food processing plants generated by the growth of biofilms on the surface of industrial pipes, and the factors and surface properties that affect the formation of biofilms on stainless steel have not been fully characterized. The adhesion and growth of bacteria on the surface of stainless steel promotes corrosion of the material, microbiological contamination, interferences with production processes by causing breakdowns and compromising the final quality of the product, healthcare diseases, and strong impact on economic losses, among other problems [2]. Biofilms can be defined as a microbial consortium embedded in a self-produced exopolymer matrix composed mainly of exopolysaccharides (EPS). It comprises microbial cells, enzymes, proteins, and nucleic acids. Microorganisms living inside this matrix benefit from nutrient and water supplies improved by lateral gene transference [3,4] and it protects them against adverse environmental conditions such as desiccation and chemicals, including detergents, disinfectants, and antibiotics [5,6]. Additionally, biofilms can operate as a reservoir for pathogens causing potential disease outbreaks [7]. Bacterial adhesion to a surface and consequent formation of a biofilm can generate severe health problems that can lead to microbial contamination and chronic infections. On the basis of reports issued by the Centers for Disease Control and Prevention (CDC) [8], foodborne diseases are a serious health problem worldwide, causing 48 million cases each year in the United States alone, with 128,000 hospitalizations and 3000 deaths, regardless of intensive efforts to improve hygienic conditions during the food production process [9]. Figure 1 shows the typical biofilm life cycle, which can be summarized in four main steps. First, bacteria become attached in a reversible way to the surface (using mechanisms such as Van der Waals forces). Then, after some time, this attachment to surface is irreversible as soon as bacteria use cellular structures designed for surface attachment (such as protein fimbriae) and they begin to produce slimy EPSs, thus colonizing the surface. In a third step, the EPS production, as well as the secretion of quorum sensing signals allows the emerging biofilm community to develop a complex three-dimensional structure, which enables an increase in the adherence between bacteria and the supporting surface (Figure 1). Finally, biofilms can propagate through detachment of small or large clumps of cells, or by a kind of “seeding dispersal” that releases individual cells [10,11,12].

Among all the strategies used to eradicate bacterial biofilms from an industrial distribution system, the best choice would consist of preventing their formation by incorporating mechanical, chemical, and thermal processes to prevent the biofilm formation as efficiently as possible. In this sense, there are many cleaning procedures, such as clean on place (CIP) or pasteurization, that are specifically designed to maintain a clean and hygienic environment, including in piping and fitting systems. The FDA (Food and Drug Administration) allows the use of sodium hypochlorite as a disinfecting agent on food industry equipment surfaces in direct contact with food, but the release of chlorine can cause pitting, resulting in surface deterioration. Other disinfecting agents are used in some food industries (sodium hydroxide, peracetic acid, detergents, benzalkonium chloride) [9,13,14]. Hence, it is possible to affirm that many problems related to microbial activity in the distribution system are due to biofilm growth as well as the persistence of pathogens [15]. For this reason, removal of biofilms from the environment of industrial distribution systems has become a critical task to be solved.

New biofilm-control methods, such as inhibition of quorum sensing (QS), enzymatic disruption, bactericidal coating, nanotechnology, and bioelectric approaches, have successfully been studied in an effort to find effective alternatives for the prevention and growth control of biofilms [16,17,18,19,20]. To date, several approaches using different alloys and antimicrobial coatings have been developed with the objective of controlling bacterial adhesion and viability on surfaces. The administration of antibiotic agents could cause resistance problems, and therefore in the past decade, surface topography has gained much importance since nano/microscale structures showed anti-adherent properties [21,22,23]. In this context, nanotechnology can provide the necessary tools for developing novel nanomaterials with enhanced antimicrobial activity [24,25]. Many innovative techniques could be applied to create new functional surfaces, for example, via novel nanostructured surface materials, surface modifications, new coatings, or painting [26,27,28].

Concerning the severe adverse impact of biofilms on many human activities, our research group carried out a novel investigation for developing different nanomaterials showing antimicrobial activity. In order to manufacture these materials, we first employed an electrochemical method in order to grow a nanostructured surface consisting in self-ordered arrays of nanoholes on 316L stainless steel. After that, we used surface functionalization techniques and physical-chemical processes in order to modify the surface of the substrate material by incorporating additional coatings on these nanoholes with antimicrobial and bactericide properties, such as Ag nanoparticles or Ag and TiO_2_ films [29,30,31,32,33,34]. The versatile techniques and processes here employed were electroless process, electrochemical deposition methods, and atomic layer deposition (ALD) technique [35,36,37].

The surface morphologies, chemical properties, stability, and optical analysis of the different functionalized materials grown on nanostructured 316L stainless steel were characterized by scanning electron microscopy (SEM), X-ray photoelectron spectroscopy (XPS), inductively coupled plasma mass spectrometry (ICP-MS), and photoluminescence spectroscopy. Furthermore, we also investigated the effects of these functional nanomaterials on biofilm growth such as *C. parapsilosis*, *E. coli*, *Ps. aeruginosa*, *S. aureus*, and *S. epidermidis.* These bacterial and fungal species were selected because in some cases their presence is usual in freshly milked milk, such as *S. aureus* [38], or are common environmental species such as *Ps. aeruginosa* [39]. In the case of some of these bacterial species, their presence in industrial pipelines and its adhesion to them allows for the subsequent union of other bacterial species, which represents a health risk, such as *S. aureus* or *Ps. aeruginosa* [40,41], whereby inhibiting the initial stages of adhesion of any bacterial cell is a key success factor when preventing the subsequent development of mixed biofilms or with the presence of potential pathogens. *Ps. aeruginosa* biofilms can contain other species, such as the pathogen *Listeria monocytogenes*, generating synergies of physical type (the extracellular matrices of the biofilms act directly as a barrier to the penetration of disinfectants and other biocidal agents) and physiological (the molecular mechanisms of resistance to disinfectants can be used for the benefit of accompanying bacteria or fungi, which generally would not possess these detoxification enzymes) [42,43].

The results here reported show that these novel functional nanomaterials display excellent antimicrobial and bactericide properties, allowing for their applicability on 316L food-grade stainless steel pipelines.

## 2. Materials and Methods

### 2.1. Nanostructuring of 316L Stainless Steel Surface

The 316L stainless steel plates (Irestal, Barcelona, Spain) were cut into 13 × 13 × 5 mm^3^ size. These materials were employed as starting substrates for the growth of nanoholes on their top surface in order to develop a nanostructure. The specific composition, in percentage by weight, of this steel in particular is shown in Table 1.

First, the stainless steel substrates were cleaned by sonication in isopropanol and ethanol. After that, the procedure for the nanohole formation on the 316L stainless steel surface was performed by following an electropolishing process in an electrolytic bath, whose temperature was maintained between 0 and 10 °C and at a constant anodic voltage of 40 V for 10 min. The electrolyte bath was composed of a mixture of 50 mL of perchloric acid and 950 mL of ethylene-glycol. Perchloric acid was used to achieve the proper low pH for promoting the ionization of metallic atoms into metallic cations instead of oxide formation. Ethylene-glycol ether ensures a high viscosity of the electrolyte. Acid and viscous electrolytes allow for a better control in the anodic conditions, creating 2 different environments along the nanohole formation, one inert at its top side and the other chemically reactive at the bottom. Thus, the inhibition of the oxide dissolution at the top side of the nanoholes leads to a less passivation of the stainless steel starting substrate, due to the less aggressive nature of the mixtured organic and acidic electrolyte [44]. The electrolytic solution was vigorously stirred by a rotating magnet because the agitation plays a crucial role in the nanoholes growth on the material surface [45].

Stainless steel samples employed as anodes were positioned vertically in front of a platinum foil counter electrode for the electrochemical polishing procedure. These 2 electrodes were linked up to a DC generator (Model Xantrex 300V 9A, Elkhart, IN, USA). After the electropolishing process, the samples were rinsed with large amounts of deionized ultrapure water (resistivity value: 18.2 MΩ/cm), then cleaned during 10 min with ethanol in an ultrasonic bath and finally dried by air.

**Figure 2:**
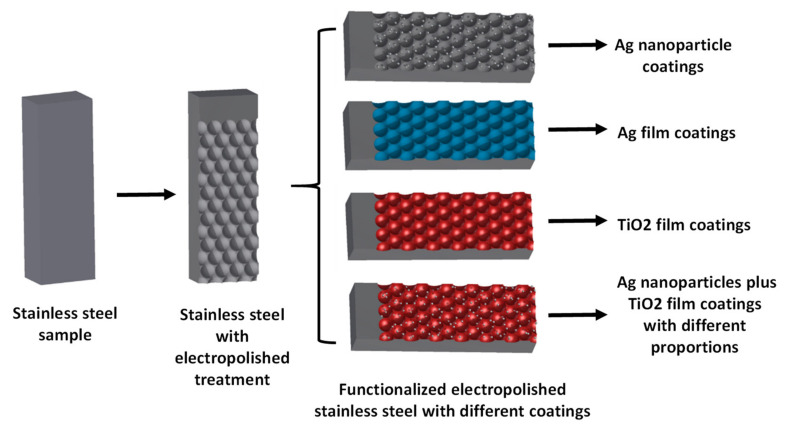
Schematic description of the nanostructuring and functionalization procedure performed on starting 316L food-grade stainless steel. Image is self-created.

Subsequently, the samples were functionalized with Ag nanoparticles, Ag films, or TiO_2_ films. These coatings were performed by means of surface modification techniques such as electroless deposition, conventional electrodeposition, or ALD, respectively, as described in detail in the following sections. Figure 2 provides a schematic illustration of the nanostructuring and functionalization procedure performed on starting 316L food-grade stainless steel.

### 2.2. Ag Nanoparticle Coating by Electroless Deposition

Coatings of functional nanostructured samples made by Ag nanoparticles (AgNPs) were deposited on the samples surface through an electroless deposition method. By means of this electrochemical process, one is able to obtain AgNPs by the reduction of [Ag(NH_3_)_2_]^+^ with glucose. 

For the preparation of [Ag(NH_3_)_2_]^+^ solution, firstly, diluted ammonia solution was dropwise added to AgNO_3_ solution until it became transparent, and after that, the final concentration of Ag^+^ was adjusted to 0.1 M by adding ultrapure water. 

The preparation of glucose solution consisted of a mixture of glucose 1 M and water.

The experimental electroless deposition procedure consisted of immersing the sample in [Ag(NH_3_)_2_]^+^ solution for 5 min. Afterwards, the substrate was dipped into DI water for several seconds and then immersed into glucose solution for 5 min. After following this process, the substrate was dipped once again into water to remove any glucose molecule. This decorating procedure was performed for several cycles to adapt the density and the size of the Ag nanoparticles deposited on the sample surface.

### 2.3. Ag Film Coating by Electrodeposition

Ag thin film electrodeposition process was carried out at room temperature under potentiostatic conditions in a two-electrode electrochemical cell equipped with an insoluble Pt mesh counter-electrode, being the sample acting as the working electrode. The deposition potential was selected at −1.7 V and the electrodeposition time was fixed to 1 min. The electrodeposition process was controlled by unit Keitley (Model 2010, Beaverton OR, USA). The electrolyte employed was a commercial silver plating solution with an Ag concentration of 28.7 g/l (Alfa Aesar).

### 2.4. TiO_2_ Film Coating by Atomic Layer Deposition

TiO_2_ thin film coating of the nanostructured stainless steel samples were performed in a Savannah 100 thermal ALD reactor from Cambridge Nanotech (Waltham, MA, USA). Titanium tetraisopropoxide (TTIP) and water were selected as precursors and Ar as the carrier gas. The exposure time was of 60 s with the purpose that the gaseous precursor could have a whole diffusion through the nanoholes of the nanostructured samples. Between 2 subsequent precursor pulses, an extended purge (90 s) with Ar flow of 50 sccm was performed in order to evacuate the excess of unreacted gaseous precursors from the ALD reaction chamber, as well as reaction by-products. The temperature in the reaction chamber was of 250 °C. The number of ALD cycles was adjusted according with the growth rates of precursor (0.05 nm/cycle), in order to adjust the thickness growth of deposited layer to around 5 nm [46].

### 2.5. Morphological Characterization of Samples

Structure and morphology of the samples were firstly checked by scanning electron microscopy (SEM, Akishima, Tokyo, Japan) in a Quanta FEG 650 microscope. Top-view SEM images were obtained for the samples’ surfaces. The SEM images were further analyzed by using ImageJ software [47] for determining the main basic lattice parameters of the samples (pore size and interpore distance). Prior to this characterization, the samples were cleaned on distilled water. Since the samples are metallic, a preparation is not necessary due to their inherent ability to conduct electricity.

### 2.6. Chemical Analysis of Samples

X-ray photoelectron spectroscopy (XPS, Berlin, Germany) was used to perform the chemical characterization of the samples. XPS measurements were performed using a Phoibos 150 with a monochromatized X-ray source (Kα Al = 1486.74 eV) at 13 kV and 300 W. The energy analyzer worked on constant pass energy mode. Survey spectra were performed using 90 eV pass energy and 1 eV step energy, and high-resolution spectra were taken with 30 eV pass energy and 0.1 eV step energy. Spatial resolution was achieved by combining a small X-ray spot size (3.5 × 1 mm^2^) with the electromagnetic lenses working in small area mode. Charge shift was compensated with a flood electron gun when necessary. Position of spurious C1s indicated that it is not necessary to correct the energy shift due to charge effects. Shirley-type base lines and 70% Gaussian–30% Lorentizan curves were used for all mathematical fittings, carried out in Casa XPS software using Marquardt–Levenberg or Simplex methods [48]. Prior to this characterization, the samples were cleaned on distilled water in order to avoid any contamination from organic origin.

### 2.7. Stability of the Samples: NP Migration Test

For a safe application of the different functionalized materials in pipelines, it is necessary to study the potential of migration of nanoparticles (NPs) from different coatings, more specifically, Ti and AgNPs, due to potential health risks by their migration. In Europe, the EFSA (European Food Safety Authority) recommends not exceeding the group-specific migration limits of 50 µg/L or 50 ppb in water. In order to investigate the potential of migration of the different ions, we employed inductively coupled plasma mass spectrometry (ICP-MS) for the detection and characterization of the ions on ultrapure water solutions in contact with 316L stainless steel substrates containing these ions on their surface. The advantages of ICP-MS technique for migration studies are: (i) its great sensitivity that allows for the detection of NPs at low mass concentrations (ppb range), (ii) the ability to quantify the ratio between ions and NPs of a certain element in migration solutions, and (iii) the possibility of direct analysis without the need for sample preparation or after simple dilution with ultrapure water.

Firstly, all substrates were immersed in ultrapure water at room temperature for 1 year. Following this, different water samples were filtered with a PTFE Filter Membrane (0.45 µm pore size) and dilutions of 1:100 in HNO_3_, 2% in volume, were prepared. The methodology here proposed involves the analysis of these dilutions by using ICP-MS Agilent Series 7700× equipment (Santa Clara, CA, USA) for this study. Helium was employed as cell gas (4.3 mL/min). Integration time was fixed to 0.2 s. The 47 Ti and 107 Ag isotopes were monitored, and Ir was used as internal standard to compensate for matrix suppression effect and improving the accuracy of measurement. MassHunter Workstation was used as data analysis software. The parameters of the method are illustrated in Table 2.

### 2.8. Optical Analysis of the Samples

Photoluminescence spectra were carried out to determine the optical properties of the samples, which were acquired using a Varian Cary Eclipse fluorimeter (Varian, Palo Alto, CA, USA), provided to Xe-900 Xenon Arc Lamp, using 5 nm slits in both excitation and emission monochromators, with a wavelength scan speed of 1200 nm·s^1^ at room temperature. Firstly, it is necessary to clean the different samples on distilled water in order to avoid any contamination from organic origin.

### 2.9. Antibiofilm Assays

Each microbial species (*C. parapsilosis* CECT 1449, *E. coli* ATCC 11775, *P. aeruginosa* ATCC 27853, *S. aureus* CECT 240, and *S. epidermidis* CECT 231) was inoculated in 3 mL TSB (tryptic soy broth; Merck, Madrid, Spain) medium without antibiotic at 37 °C overnight in order to obtain the corresponding preinoculum. These cultures were used for preparing a working solution of 2 mL TSB with 107 colony-forming units (CFU)/mL on the basis of the DO600 of the preinoculum for each species. Then, 12-well microtiter plates were used for carrying out the antibiofilm assays. Following this, 4 replicas from each metallic probe were deposited in the bottom of the corresponding 4 wells, and 2 mL with the microbial cells were added to each well. Four control metallic probes were deposited in 4 wells containing only sterile TSB medium as negative control. Microtiter plates were covered with Parafilm to avoid evaporation and incubated at 37 °C for 48 h at 115 rpm. Then, metallic probes were transferred to a new microtiter plate and washed 3 times with 5 mL phosphate-buffered saline pH = 7.4 (PBS, KH_2_PO_4_ 144 mg/L, NaCl 9 g/L, Na_2_HPO_4_-7H_2_O 975 mg/L). Next, 1–1.5 mL of 2% agarose solution was used for immersing the metallic probes, leaving the upper treated surface of each probe uncovered, wherein the microbial biofilm was eventually present. Finally, 150 µL of the BacTiter-Glo reagent solution (BacTiter-Glo™ Microbial Cell Viability Assay, Promega, Madrid, Spain) was added to the surface of each metallic probe, including the negative controls, and incubated for 15 min at room temperature in darkness. This reagent contains enzymes in charge of cell lysis, as well as luciferin substrate and luciferase, which provide luminescence in the presence of ATP (from lysed viable microbial cells). The 150 µL was recovered from the metallic probe surface and deposited in a well of a 96-well black microtiter plate. The obtained luminescence was measured in a luminometer (GloMax Multi, Promega, Madrid, Spain) and it was therefore proportional to the amount ATP and viable cells in the surface biofilm. A calibration curve was created for each microorganism using known cellular concentrations in black microtiter wells (10^8^, 10^7^, 10^6^, 10^5^, 10^4^, 10^3^, 10^2^ CFU/mL), incubated in the same way with the luminescence reagent in order to relate this parameter with cellular density. A total of 8 different metal probe types were used in these experiments. The characteristics of these samples can be seen in Table 3.

### 2.10. Statistical Analyses

Data are expressed as the mean value ± S.E.M. Statistical analyses were conducted using ANOVA test when the quantitative data presented normality and the variances were assumed equal. Normality was analyzed using the Shapiro–Wilk test. In the absence of normality, the Kruskal–Wallis test was used. Graphics were carried out using GraphPad Prism software (version 8, GraphPad Software, San Diego, CA, USA). In all cases, a *p*-value < 0.05 was considered statistically significant.

## 3. Results and Discussion

### 3.1. Samples Microstructure

**Figure 3:**
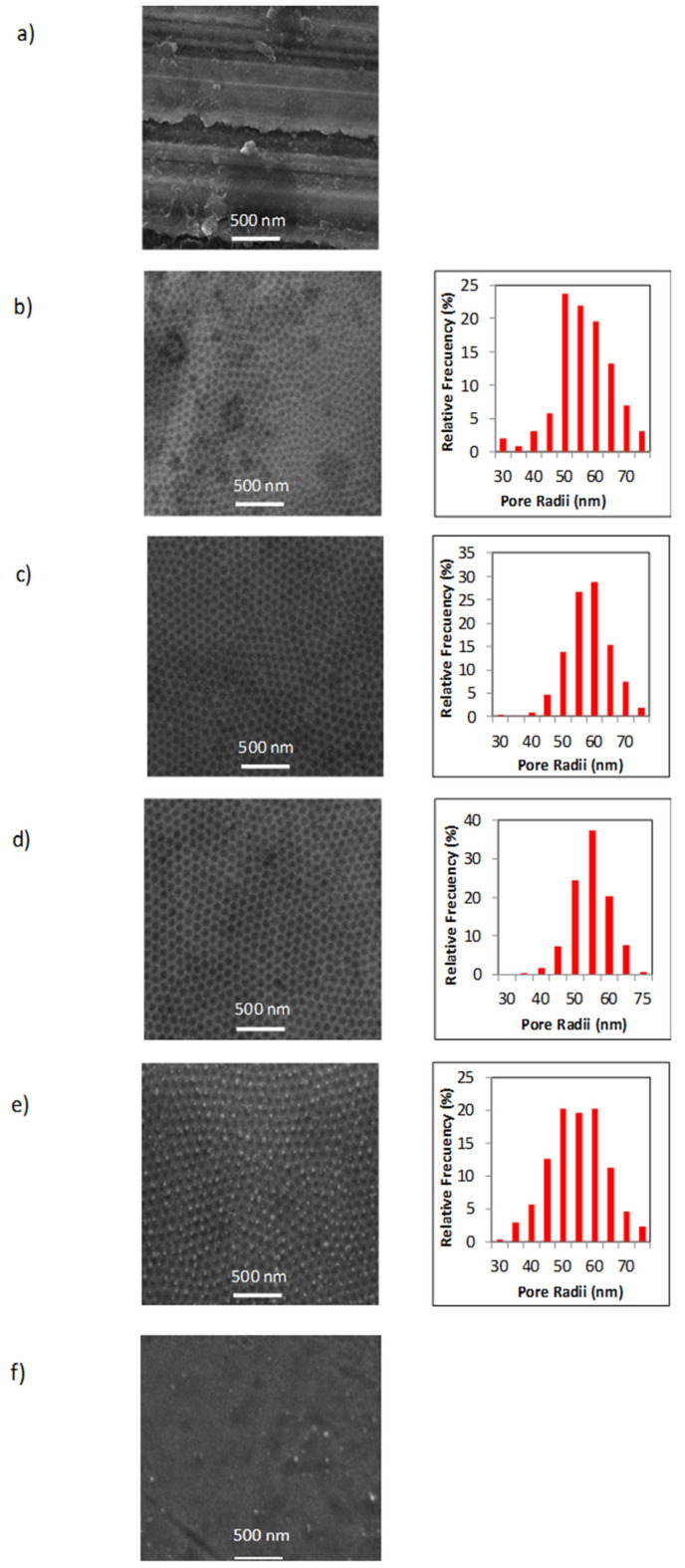
SEM top surface views corresponding to the sample S1 (**a**), sample S2 (**b**), sample S3 (**c**), sample S4 (**d**), sample S5 (**e**), and samples with AgNPs plus TiO_2_ films with different proportions (samples S6-S8) (**f**). The respective histograms show the pore radii distribution obtained from each SEM micrograph, ranging between 50 and 60 nm.

Figure 3 shows the SEM top-view micrographs taken for all the samples. Figure 3a corresponds to untreated as-obtained 316L stainless steel sample (sample type S1), while Figure 3b corresponds to 316L stainless steel with nanoholes induced by the electropolishing process (sample type S2). This figure shows that the nanohole arrangement exhibited a highly hexagonal self-ordered structure in a similar way to the one obtained in nanoporous anodic alumina [49].

SEM images of nanostructured stainless steel samples, exhibiting the functionalized nanoholes with different coatings deposited inside, are shown in Figure 3c-f. From these micrographs, one can note that the sample surface changed depending on the coating type. In the case of the samples that were coated with Ag (Figure 3c,d), it can be noted that their micrographs were better defined than the sample without functionalized nanoholes. This fact was due to their better conductivity by the presence of Ag metallic coatings. Samples coated with TiO_2_ film by ALD showed the TiO_2_ nanoparticles homogeneously distributed over the whole surface (Figure 3e), and in the cases of the samples with TiO_2_ films plus AgNPs by electroless, one is able to see that all nanostructured surface was fully covered (Figure 3f). 

On the other hand, the pore radii dispersion histograms shown for all figures constitute clear evidence of the narrow size distribution of around 50–60 nm for the nanoholes in the arrays.

### 3.2. Chemical Composition of Samples

In order to obtain detailed information about the surface chemistry of the samples, we performed a XPS analysis. Several samples were selected: Ag nanoparticle coating by electroless (sample S3), ED Ag film coating (sample S4), TiO_2_ film coating (sample S5), and TiO_2_ film plus electroless plated Ag coating (sample S6).

**Figure 4:**
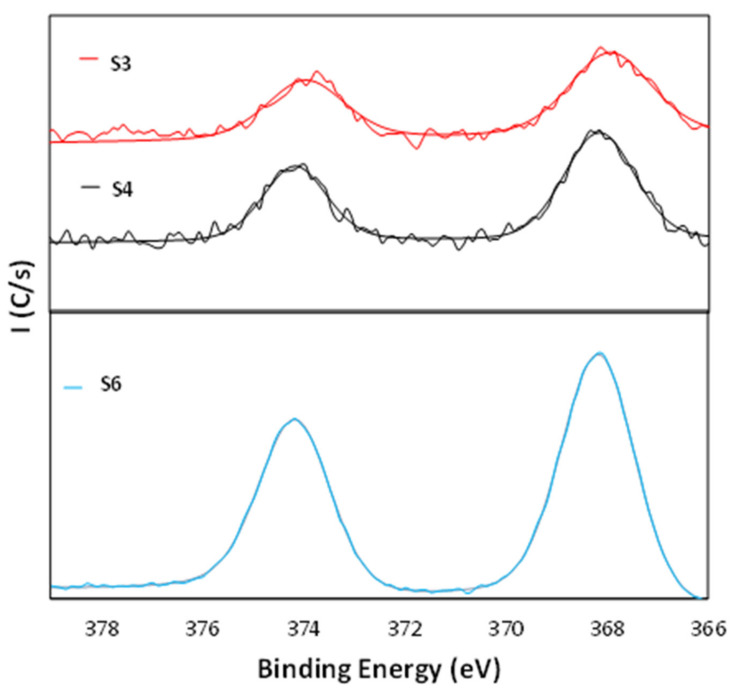
Ag 3d core level XPS spectra for the samples S3, S4, and S6.

Figure 4 shows the Ag 3d core level spectra for the Ag samples. Sample S3 showed two peaks at 368.0 eV and at 374.5 eV. These values are in good agreement with previously published XPS data for Ag 3d, where the binding energies of Ag(3d5/2) and Ag(3d3/2) peaks are located at 368.2 and 374.0 eV, respectively [50,51,52]. The sample S4 showed two peaks at 368.2 eV and at 374.7 eV. Just as previously noted, these energy values are in good agreement with previously published XPS data for Ag 3d. The sample S6 showed two peaks at 368.0 and at 374.0 eV, but in this sample, it is possible also to note an increase on the value of the intensity peaks. This fact is due to this sample having a higher Ag content than the other samples with Ag coatings. An explanation of this fact is that TiO_2_ surface has a higher surface charge density and the TiO_2_ surface can be activated by ammonia to produce the reactive Ti–O group. This means that a silver ammine complex [Ag(NH_3_)_2_]^+^ can be attracted to the partially negatively charged TiO_2_ surface, and therefore it possibly forms a strong chemical bond between the Ti–O group and elemental Ag on the TiO_2_ surface [53]. Therefore, it could be concluded that the TiO_2_ surface layer improved the adhesion of the electroless-plated silver layer on the surfaces and assisted a continuous and uniform coating of the silver layer.

**Figure 5:**
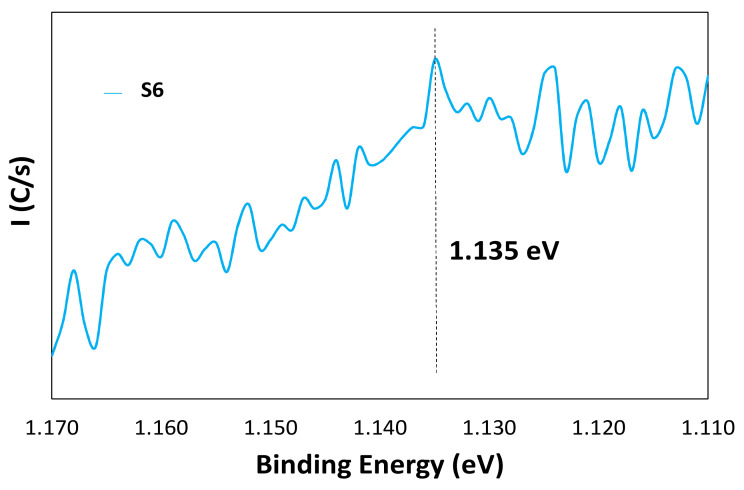
Ag M5NN Auger spectrum obtained from the sample S6.

To strengthen this, Figure 5 shows the Ag M5NN Auger spectrum obtained from the sample S6. The maximum appeared at a binding energy of 1135.0 eV (351.6 eV kinetic energy), a value that is associated in the literature to Ag metal [50,51,52].

**Figure 6:**
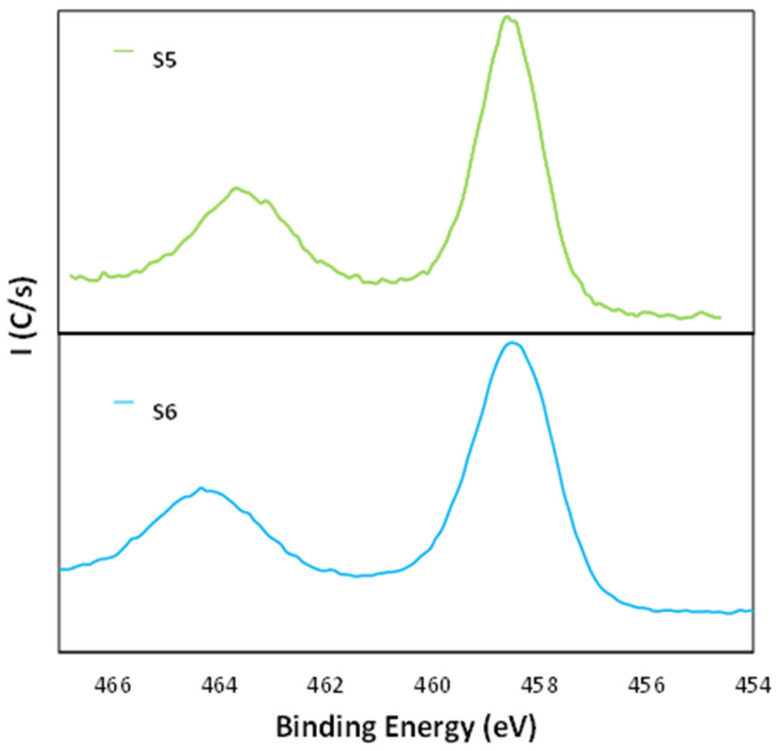
Ti2p core level XPS spectra for the samples S5 and S6.

Figure 6 shows the Ti2p core level spectra for the samples S5 and S6. The sample S5 exhibited two peaks at 458.5 eV and at 464.1 eV, whereas the sample S6 exhibited two peaks at 458.5 eV and at 464.2. The indexation of these peaks can be derived from the literature, where the peaks at 454.7 eV and at 460.6 eV are ascribed to TiC structure [54] while the peaks at 458.3 eV and at 464 eV correspond to TiO_2_ structure [55]. Moreover, both samples exhibited the Ti 2p3/2 peak at 458.5 eV and a separation of 5.7 eV with respect to the doublet, which is also characteristic of the titanium oxide [56,57]. Therefore, it can be concluded that the structures correspond to the TiO_2_ type.

**Figure 7:**
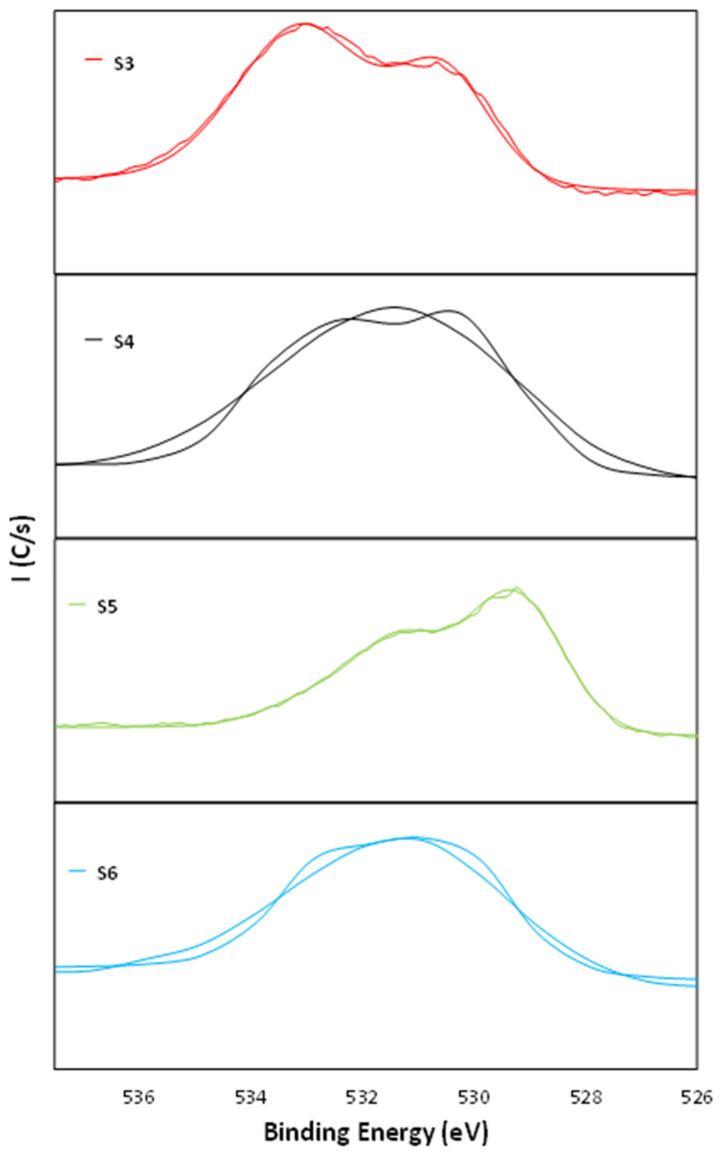
XPS spectra with different contributions of O1s for the samples S3, S4, S5, and S6.

In addition, Figure 7 shows the O1s spectra with different contributions. In the case of samples S3 and S4, two bands appeared at 530.0 and 532.1 eV, which were due to contributions from Fe_2_O_3_ from the steel and C=O groups, respectively [50,51,52]. For the samples S5 and S6, the bands appeared at 529.9 and 532.0 eV from TiO_2_ and organic C=O, respectively, which were assigned to oxygen bounded to tetravalent Ti ions [58].

### 3.3. Stability of the Samples: NP Migration Test

Migration of Ti and Ag ions from different coatings was determined by immersion of samples into distilled water at room temperature for one year, and the concentrations of the ions on water were determined by inductively coupled plasma mass spectrometry (ICP-MS). The obtained results are shown in Table 4. 

Ti ions showed a very low migration potential in comparison with Ag ions, which indicates that TiO_2_ films coatings were very stable in the experimental conditions studied and the use of these coating types is safe for human health (the overall migration for all samples was below 50 µg/L or 50 ppb on water) [59].

For the case of Ag ions, we can see that migration was much more noticeable because some of the samples reached values close to 1400 ppb, well above of the values established as the limit by the competent authorities (50 µg/L or 50 ppb on water). Therefore, among the samples analyzed that had Ag coatings, only the sample S8 complied with the legal regulations. However, one important conclusion that can be drawn is that Ag ion concentration on water may have been reduced with the use of TiO_2_ as fixing agent, and it can also be seen that the TiO_2_/AgNP ratio deposited on substrates’ surfaces would play an important role on the fixation of the AgNPs (it is optimized if the Ag content increased in this ratio, with sample S8 having more Ag than the rest of the samples). These results indicated that the TiO_2_/AgNP ratio used can act as a beneficial effect in the reduction of the migration of silver nanoparticles.

### 3.4. Optical Analysis of the Samples

**Figure 8:**
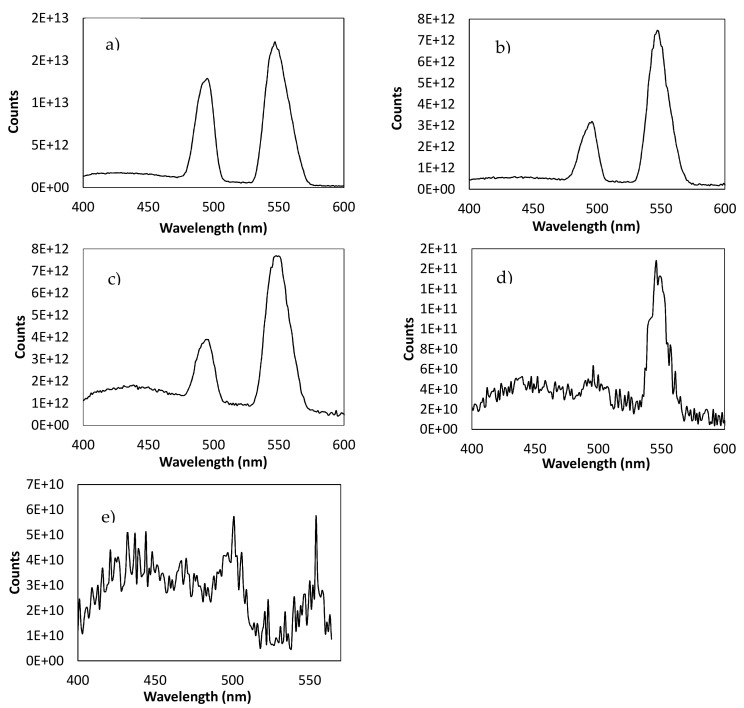
PL emission spectra of the samples functionalized with different coatings: (**a**) EP steel with Ag coatings (samples S3–S4); (**b**) EP steel with TiO_2_ thin films (sample S5); (**c**) EP steel with double TiO_2_ films plus electroless plated Ag coating (sample S7); (**d**) EP steel with TiO_2_ films plus electroless plated Ag coating (sample S6); and (**e**) EP steel with TiO_2_ films plus double electroless plated Ag coating (sample S8).

Photoluminescence spectroscopy (PL) is a very useful spectroscopic method to determine the optical properties and the energy gap of the coatings that had the different samples, and thus only the samples that had coatings in their surface were selected in this study. The PL emission spectra that were obtained at room temperature with an excitation wavelength of 370 nm are shown in Figure 8.

Figure 8a shows the PL spectrum corresponding to the samples S3–S4. In these samples two kinds of PL emission peaks were observed. These two fluorescence emission peaks were attributed to relaxation from the electronic motion of surface plasmon, and a recombination of sp electrons with holes in the d band, respectively [60].

Figure 8b shows the PL spectrum corresponding to the sample S5. The spectrum shows two peaks that correspond to anatase TiO_2_. The peaks observed at 497 and 549 nm were associated with the oxygen vacancies on the surface of the TiO_2_ film. The peak at the value of 497 nm was the neutral oxygen vacancy, while the one at 549 nm was the oxygen vacancy losing an electron [61].

Figure 8c,d shows the PL spectrum corresponding to the samples coated with TiO_2_ films plus different ratios of AgNP coating (samples S6–S8). Relying on these figures, one can see that the peak intensity of PL spectrum decreased with the increasing of the Ag content, which confirms the ratio decrease of electron hole recombination in the presence of light illumination [62]. In all of these cases, the introduction of transition metal ions into the TiO_2_ lattice caused a decreasing trend in the PL intensities as a result of a shorter distance corresponding to the inter-band metal ions that could lead to energy transfer among nearby ions.

The other aim of the study was to analyze the change in the energy band-gap between electronic states of the samples depending on functional coating onto their surfaces because the excitation of semiconductor nanoparticles takes place when hν_exc_ ≥ E_conduction_ – E_valence_. Thus, there is a minimum energy (gap energy), Eg, required to promote an electron from the valence band to the conduction band and its value is ascribed to the surface defects originated by the functionalization treatments.

From the analysis of these photoluminescence spectra, we estimated the energy of the forbidden band (*E_g_*), indirect band gap, using Tauc’s law for direct transitions [63]:

(1) $$\left(\alpha h\nu^{2}\right)=k\left(h\nu -E_{g}\right)$$

where *α* is the absorption coefficient, *hν* is the energy of the incident light, and *k* is a constant. According to Valeur and Berberan-Santos [64], the intensity of photoluminescence is proportional to the absorption coefficient by

(2) $$F≅2.3K_{P}\;.\;\;{ø}_{P}\;.\;\;I_{O}\;.\;\alpha \;.\;b\;.\;c$$

where *K_p_* is a constant depending on several instrumental parameters, *ø_p_* is the PL quantum yield, *I_0_* is the intensity of incident light, *b* is the optical path, and c is the concentration of the luminophore. Therefore, Equation (1) can be transformed into Equation (3):

(3) $$\left(Fh\nu^{2}\right)=k´\left(h\nu -E_{g}\right)$$

and then *E_g_* can be estimated from the graphical representation of *(Fhν)^2^ vs*. *hν* by extrapolating the straight line to (*Fhν*)^2^ = 0. The obtained values for *E_g_* are summarized in Table 5.

In Table 5, it is possible to see that the optical band gap decreased as the Ag dopant concentration increased (S7 < S6 < S8). The modification of band gap was essentially due to the interaction between the Ag and TiO_2_ structures. Therefore, Ag nanoparticles caused the creation of trap levels between the conduction band and valence band of TiO_2_ for the strong covalent interaction and connectivity between AgNPs with TiO_2_, which was reflected in a significant band gap reduction [62].

### 3.5. Antibiofilm Assays

Antibiofilm tests were performed as described above to analyze the antimicrobial activity of the different nanomaterials. For this, one fungal species (*C. parapsilosis* CECT 1449) and five bacterial species (*E. coli* ATCC 11775, *Ps. aeruginosa* ATCC 27853, *S. aureus* CECT 240, and *S. epidermidis* CECT 231), usually found in biofilms associated with the agri-food industry, were used. As negative control, the strain *B. cereus* CECT 131, which does not generate biofilm, was used as reference for the analytical method on biofilm development (in order to detect the basal levels due to the presence of ATP from living attached cells, measured via a luminescence reaction). The obtained calibration curves for each one of the microorganisms are shown in the Supplementary Materials (Figure S1). 

**Figure 9:**
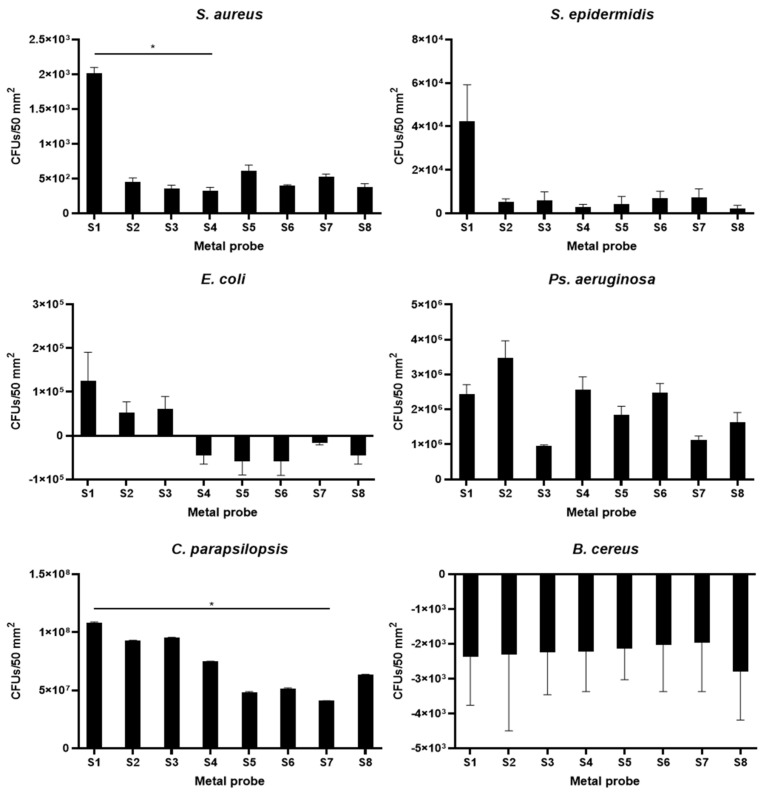
CFU values obtained after the biofilm assays on the 8 different types of metal probes: (**a**) *S. aureus*, (**b**) *S. epidermidis,* (**c**) *E. coli*, (**d**) *Ps. aeruginosa*, (**e**) *C. parapsilosis*, (**f**) *B. cereus*. Statistically significant differences (ANOVA test, *p*-value < 0.05) are marked with an asterisk: *p*-value for *S. aureus* S1–S4 comparison is 0.0440, *p*-value for *C. parapsilosis* S1–S7 comparison is 0.0229.

In total, one type of control stainless steel probes and seven types of surface treatments were used in these biofilm assays. Figure 9 shows the average CFU values for each microorganism associated with the corresponding bioluminescence obtained with the two replicas of each metal probe type. Statistically significant differences are marked in Figure 9, using ANOVA test for comparing control metal probe (S1) with the different modified substrates (S2 to S8).

Figure 9 shows the effect of each treatment on different biofilm-forming microorganisms. As a general rule, a tendency in the reduction in cell viability can be observed in *S. aureus*, *S. epidermidis*, and *E. coli*, as well as to a lesser extent in *C. parapsilosis* when the surface has undergone a nanostructuration treatment (treatment types 2–8) as opposed to the untreated case (treatment type 1). However, statistically significant differences were observed in the case of *S. aureus* between samples S1 and S4 (*p*-value 0.0440), and in the case of *C. parapsilosis*, between samples S1 and S7 (*p*-value 0.0229). This phenomenon is in accordance with the literature, and it owes its explanation to the large amount of nanopores generated on the surface of the stainless steel surface. When the features of the metal surface reach a nanometric level, the real contact surface of the material with the microorganisms increases dramatically in a theoretical way, but since the generated nanopores are smaller than the microorganisms size, they grow suspended over the pores instead of confined inside them, and thus the real contact surface between the microorganisms and the metallic surface becomes smaller. Hence, the effective physical bacterial contact area on a nanostructured surface is smaller than on a non-nanostructured one, resulting in weaker overall adhesion forces, which leads to the suppression of bacterial mechanisms against antibiotics, among others. In addition, it is also thought that air trapped in the cavities may add to the effect of reducing bacterial adhesion. This assumption explains the loss of adhesion in sample types S2-S8 in comparison with the control without electropolished treatment [21,23]. However, this behavior does not seem to be met in the case of *Ps. aeruginosa*, where the nanostructure of the material alone was not enough to reduce the formation of bacterial biofilm. Since *Ps. aeruginosa* is known to produce a high amount of EPS, this could contribute to the strong binding and stabilization of cells to the surface [65]. 

As far as *C. parapsilosis* biofilm is concerned, the nanostructuration is not enough to reduce biofilm formation, probably due to the bigger cellular size of this unicellular eukaryotic microorganism, which allows for the establishment of enough contact points on the nanostructured metal surface, and additional antimicrobial coatings are needed. The most effective surface treatments in the case of yeast (*C. parapsilosis*) were the ones corresponding to the samples S5, S6, S7 (*p*-value 0.0229), and S8. These results confirm the advantages of using TiO_2_ and Ag coatings in association over nanostructured 316L stainless steel in order to inhibit the growth of all types of biofilms investigated in this study (S6 to S8). Silver coatings have been successfully used as a control mechanism against diverse *Candida* species on glass and plastic surfaces, with 22% to 34% biofilm reduction, depending on the species [66]. This behavior can be explained on the basis of the band gap reduction of the TiO_2_ due to the incorporation of AgNPs. As a result of this, the metal may inject electrons into the semiconductor, creating points defects with partially located electrons and holes on its surface. This involves the formation of superoxides, hydrogen ions (H+), and/or OOH* radicals, a consequence of electron-hole reactions, causing protein inactivation and cellular death (apoptosis) [67,68]. Consequently, it can be said that this association can be extremely effective in preventing biofilm formation and decreasing the number of bacteria [69].

## 4. Conclusions

In this study, we have described the performance of a nanostructuring and functional process that develops a potential antibacterial effect with applicability on 316L food-grade stainless steel pipelines. An environmentally friendly, low-cost, and simple yet reliable method, which can give reproducible results in industrial environment, was demonstrated. This method offers opportunities to develop antibacterial surfaces for potentially preventing bacterial infection in the food industry. According to the previously exposed results, we can establish that an appropriate selection of surface nanostructuration and functionalization treatments is of vital importance so as to have an effective control in the biofilm formation on 316L food-grade stainless steel pipelines, especially in the case of Gram-positive bacteria (*S. aureus*, *S. epidermidis*) and in *E. coli*. It can also be said that the combination of Ag NPs together with TiO_2_ thin layer deposited over a nanostructured 316L stainless steel is of particular interest to inhibit the growth of all types of biofilms investigated in this study, as this combination causes protein inactivation and cellular death, while it has become possible, at the same time, to perform an appropriated combination between these two elements to be safe for human health.

Through this research, we have demonstrated the feasibility of achieving the control of bacteria–surface interactions via surface functional modifications. This approach can provide exciting solutions to biofilm control in many research areas that directly affect human health and life, including the food industry, water treatment, medicine, or marine applications, to name a few, opening new possibilities to understand the physical interaction role of main antimicrobial mechanisms. Another important point seeking an answer lies in the fact that the real application of nanotechnology in the industry is limited at present due to the high cost of the different fabrication procedures employed for the development of these nanostructured materials [70]. However, in our study, we have demonstrated that it is possible to use this nanotechnology in 316L food-grade stainless steel pipelines by employing low-cost processes. Moreover, we analyzed the performance of these new combined materials (Ag and TiO_2_) for the food industry, representing a novel strategy of development of these nanomaterials for their use in the industry.

## Figures and Tables

**Table 1:** Compositional analysis of the 316L stainless steel plates.

Element	Fe	C	Si	Mn	Ni	Cr	Mo	N	S	P
% weight	balance	0.018	0.48	1.34	10.03	16.57	2.00	0.038	0.002	0.029

**Table 2:** Parameters of the method.

Inductively Coupled Plasma	Mass Spectrometer
RF power (W) 1550	Sampling cone nickel
Carrier gas (L/min) 1.07	Skimmer cone nickel
Plasma gas (L/min) 15.0	Peak Pattern 1 points
Sample depth (mm) 10.0	Replicates 3
Nebulizer pump (rps) 0.10	Sweeps/replicates 100
Nebulizer MicroMist	Integration time/mass 0.2 s/ion

**Table 3:** Sample type employed for the different antibiofilm assays.

Sample Type	Characteristics
S1	Negative control, unmodified stainless steel
S2	Stainless steel with electropolished treatment (EP steel)
S3	EP steel with electroless plated Ag coating (Ag nanoparticle coatings)
S4	EP steel with Ag electrodeposited (Ag film coating)
S5	EP steel with TiO_2_ thin films grown by atomic layer deposition (TiO_2_ film coating)
S6	EP steel with TiO_2_ films plus electroless plated Ag coating
S7	EP steel with double TiO_2_ films plus electroless plated Ag coating
S8	EP steel with TiO_2_ films plus double electroless plated Ag coating

**Table 4:** Ion migration values from different sample coatings.

Sample Name	47 Ti		107 Ag	
	Conc. (ppb)	Conc. RSD	Conc. (ppb)	Conc. RSD
S1	3.5	2.6	3.2	0.4
S2	4.0	3.4	5.1	0.7
S3	4.6	1.7	382.7	3.2
S4	0.1	5.5	0.0	1.4
S5	5.6	1	1.8	0.7
S6	4.0	11.2	1457.4	3.1
S7	4.6	9.4	349.6	1.2
S8	7.1	8.1	17.5	0.5

**Table 5:** Bandgap values of different samples.

Sample	*E_g_*, Energy Band Gap (eV)
S3	2.2 ± 0.1
S5	2.15 ± 0.02
S7	2.15 ± 0.02
S6	2.1 ± 0.1
S8	2.0 ± 0.1

## Data Availability

Not applicable.

## Supplementary Materials

The following are available online at https://www.mdpi.com/article/10.3390/nano11041055/s1, Figure S1: Calibration curves obtained as reference of luminescence values associated to a given cellular density for the species: (a) *S. aureus*, (b) *S. epidermidis*, (c) *E. coli*, (d) *Ps. aeruginosa*, (e) *C. parapsilosis*, (f) *B. cereus.*
