# Supplementary material for: Functional Antimicrobial Surface Coatings Deposited onto Nanostructured 316L Food-Grade Stainless Steel
Source: Nanomaterials (Basel). 2021 Apr 20;11(4):1055. doi: 10.3390/nano11041055 (PMC8074267; doi:10.3390/nano11041055)
Supplement: Supplementary file 1 [file nanomaterials-11-01055-s001.zip › nanomaterials-1179820-supplementary.pdf]

Supplementary Materials:

Functional antimicrobial surface coatings deposited onto nanostructured 316L food grade stainless steel

Authors: Gonzalez A. S., Riego Á., Vega V., García J., Galié S., Gutiérrez-del-Río I., Martínez de Yuso M. V., Villar C. J., Lombó F., Prida V. M.

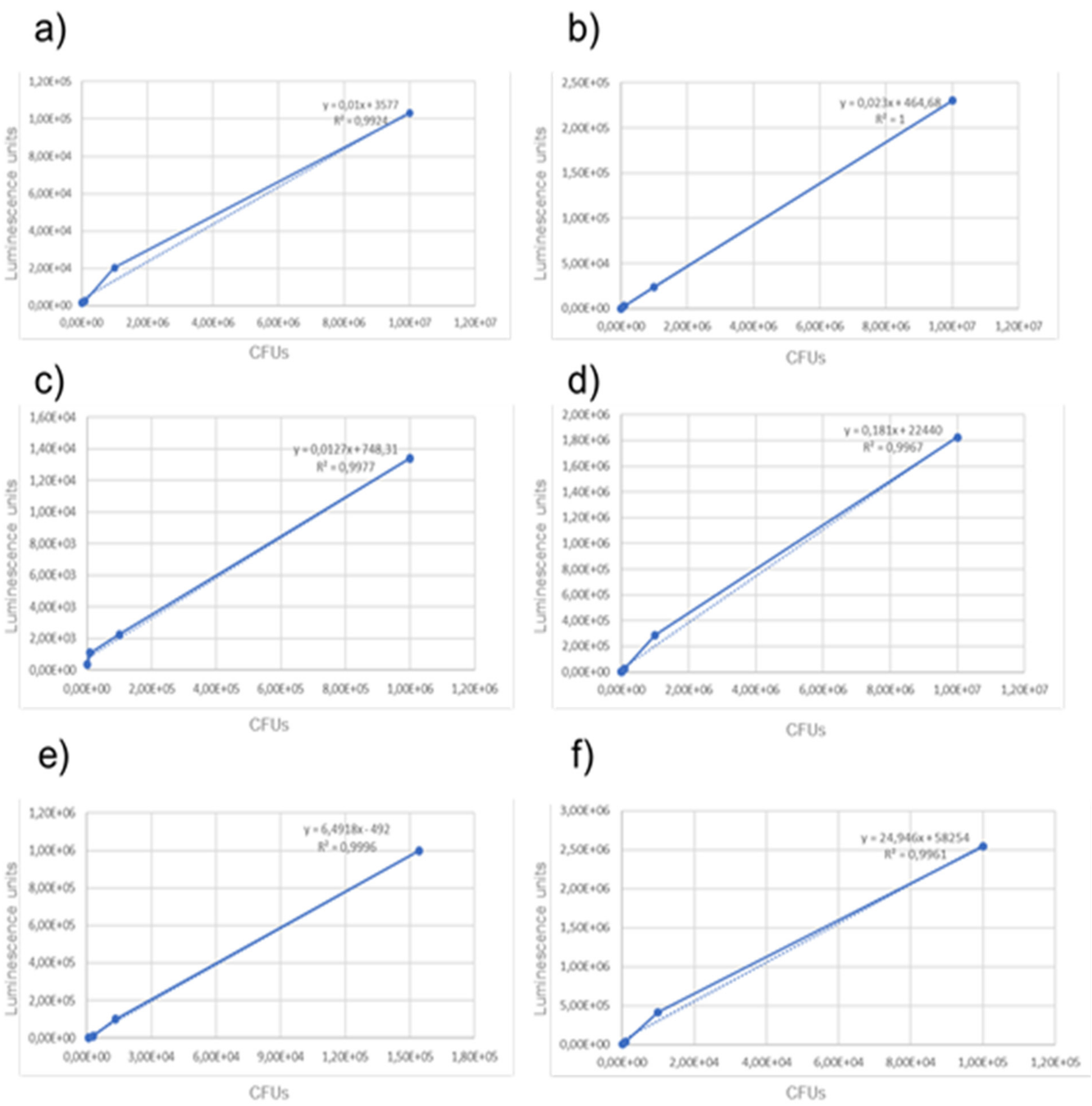

Figure S1. Calibration curves obtained as reference of luminescence values associated to a given cellular density for the species: **a**) S. aureus, **b**) S. epidermidis, **c**) E. coli, **d**) Ps. aeruginosa, **e**) C. parapsilosis, **f**) B. cereus.
